# Supplementary material for: Transparent multispectral photodetectors mimicking the human visual system
Source: Nat Commun. 2019 Nov 1;10:4982. doi: 10.1038/s41467-019-12899-8 (PMC6825164; doi:10.1038/s41467-019-12899-8)
Supplement: Supplementary file 1 — Supplementary Information [file 41467_2019_12899_MOESM1_ESM.docx]

­­­Supplementary Information

**­­­­­Transparent Multispectral Photodetectors Mimicking the Human Visual System**

Qitong Li^1^, Jorik van de Groep^1^, Yifei Wang^1^, Pieter G. Kik^1, 2^, Mark L. Brongersma^1*^

1. *Geballe Laboratory for Advanced Materials, Stanford University, Stanford, USA*
2. *CREOL, The College of Optics and Photonics, University of Central Florida, Orlando, USA*

** Email: brongersma@stanford.edu*

**Supplementary Note 1: Optical eigen modes in a single Si NW.**

High refractive index nanowires (NWs) support leaky-mode resonances due to the strong light confinement intuitively by total internal reflection [1]. Mathematically, we can apply classical waveguide theory to describe optical eigenmodes in a single Si NW [2]. The eigen mode field distribution satisfies the Helmholtz equations:

$\nabla^{2}E+{\omega^{2}\mu}_{0}\varepsilon_{0}\varepsilon\left( r \right)E=0$ (1)

$\nabla^{2}H+{\omega^{2}\mu}_{0}\varepsilon_{0}\varepsilon\left( r \right)H=0$ (2)

Where *ε(r)* = *ε*_si_ inside the NW and *ε(r)* = *ε*_air_ outside in NW. We note that in the absence of loss, *ω* can be either a real number (guided mode) or a complex number (leaky mode).

Standard boundary conditions apply at the interface between the Si NW and air:

$E_{\parallel, si}=E_{\parallel, air}; H_{\parallel, si}=H_{\parallel, air}$ (3)

$\varepsilon_{\mathrm{si}}E_{\perp, si}={\varepsilon_{\mathrm{air}}E}_{\perp,air}; H_{\perp, si}=H_{\perp,air}$ (4)

For a cylindrical single NW, analytical solutions can be found [2]. For the lowest order TM_11_ mode, we have

Inside NW:

$E_{z}(r)=C\frac{J_{0}(\kappa r)}{J_{0}(\kappa a)}$ (5)

Outside NW:

$E_{z}(r)=C\frac{H_{0}(\gamma r)}{H_{0}(\gamma a)}$ (6)

Where

$\kappa^{2}={\omega^{2}\mu}_{0}\varepsilon_{0}\varepsilon_{\mathrm{Si}}$ (7)

$\gamma^{2}={\omega^{2}\mu}_{0}\varepsilon_{0}\varepsilon_{\mathrm{air}}$ (8)

*J*_0_ and *H*_0_ represent zero-order Bessel function (standing wave) and zero-order Hankel function (propagation wave, *E*_z_ ∝ 1/*√r*), respectively. *ω* is determined by solving the following equation derived from boundary conditions:

$\varepsilon_{\mathrm{Si}}\frac{J_{0}'(\kappa a)}{J_{0}(\kappa a)}=\varepsilon_{\mathrm{air}}\frac{H_{0}'(\gamma a)}{H_{0}(\gamma a)}$ (9)

Thus, we verify that for the TM_11_ mode, a propagation wave outside the NW exists, which is a preliminary condition to show far-field coupling between NWs, as we discuss later.

For a rectangular cross-section NW, no simple analytical solution can be found. However, we can still numerically calculate the eigenfrequency and eigenmode distribution using the commercial frequency-domain Maxwell equation solver COMSOL. Firstly, we find various optical eigenmodes supported by a single Si NW suspended in the air as shown in Supplementary Fig. 1a. Then, we check if we could engineer the cross-section geometry to tune TM_11_ and TM_12_ mode such that we achieve degeneracy (Supplementary Figs. 1b,c). As we expect based on the discussion in the main text, no degenerate optical modes are found by sweeping the aspect ratio of single NW, indicating that a new mechanism must be found to realize degenerate optical resonances to realize a transparent meta-photodetector.


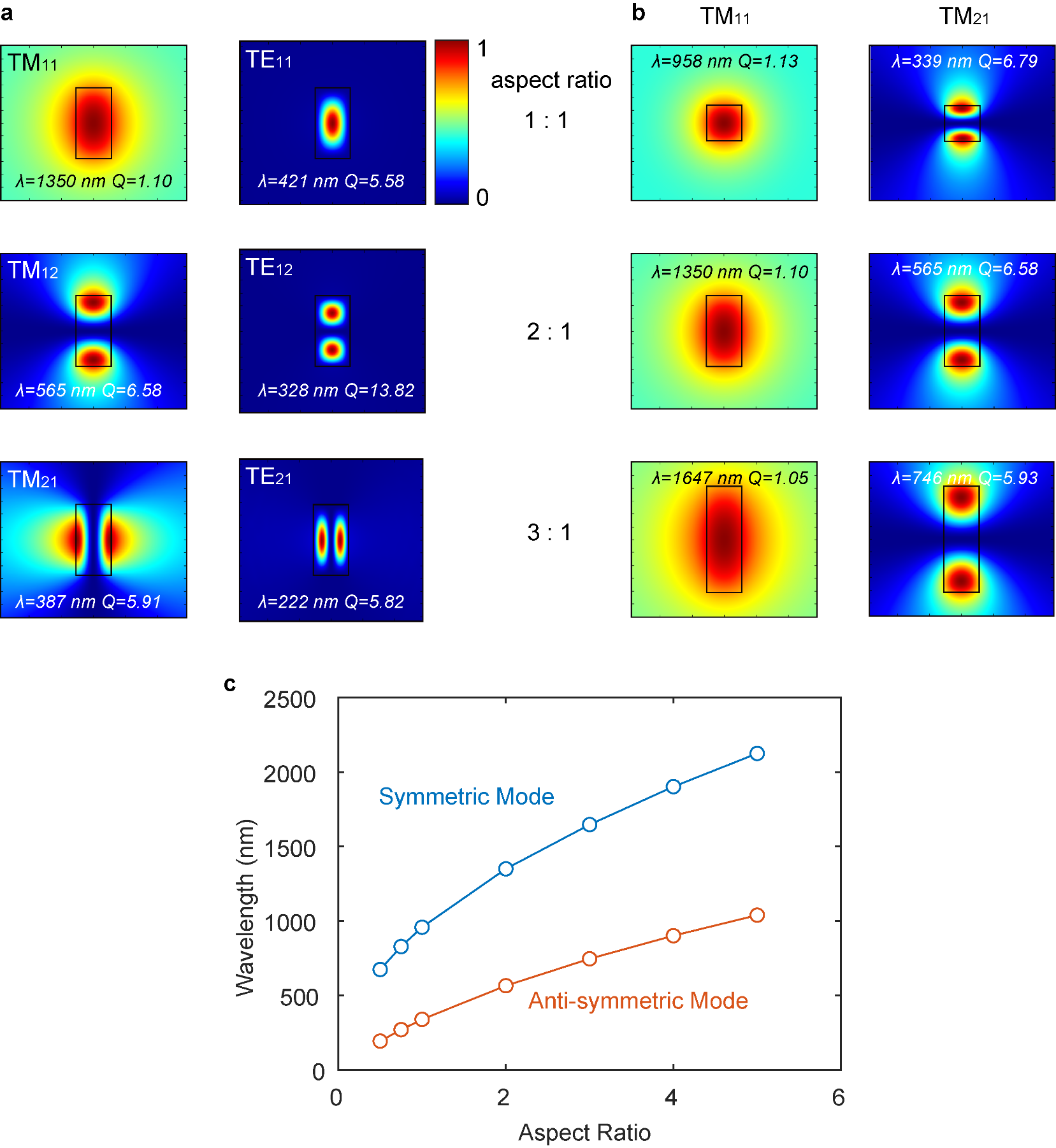


**Supplementary Figure 1.** (a) Square of modulus of electric (left side, $\left| E_{z} \right|^{2}$) / magnetic (right side, $\left| H_{z} \right|^{2}$) field distribution for various eigen modes of a single Si NW (height = 110 nm, width = 55 nm). (b) Square of modulus of electric field distribution ($\left| E_{z} \right|^{2}$) for first-order (TM_11_) and second-order (TM_12_) optical resonances of a single NW. The width of NW is fixed as 55 nm and the aspect ratio of NW is tuned from 1 to 3. (c) The center wavelength of the eigen symmetric and anti-symmetric modes as a function of the aspect ratio of a single Si NW. The width of the NW is fixed as 50 nm.

**Supplementary Note 2: Modified first-order optical resonance through far-field coupling.**

From Supplementary Section 1, we know that the TM_11_ mode supports strong far-field radiation (*E_sca_*~1/*√r*). The light radiated from the NWs in the plane of the NW array can produce a standing wave in the gap between the NWs, where it can increase the field intensity. In a simple picture, the scattered light from one NW in the array radiates to its neighbors and they scatter it back to the original NW. This NWs then can re-scatter again and the entire cycle repeats itself. A resonance occurs when the oscillating light picks up a phase equal to an integer number of times 2π. This results in a Fabry-Pérot-like resonance condition:

$2kL+2\varphi_{R}=2n\pi$ (10)

Where *k* is the free-space wavevector, *L* is the length of the resonator, and *φ_R_* is the scattering phase pick-up by the NWs. Thus, the resonant wavelength should have a quasi-linear relation to the period of the NW array, as shown in Supplementary Fig. 2. This verifies that the first-order optical resonance in a NW array can be tuned without reshaping the NW. This is helpful in the design of our Spectropolarimeter as typically the resonances in high index semiconductors are fully controlled by their size and shape. Here, it provides an extra degree of freedom to spectrally control and align different optical resonances. We emphasize that the coupling happens between adjacent NWs and thus we don’t need a large NW array to observe the resonant effect, as confirmed by the 2-μm sized pixels used in Fig. 2c. This is ideal since a smaller pixel size is preferred for practical applications to achieve higher spatial resolution.


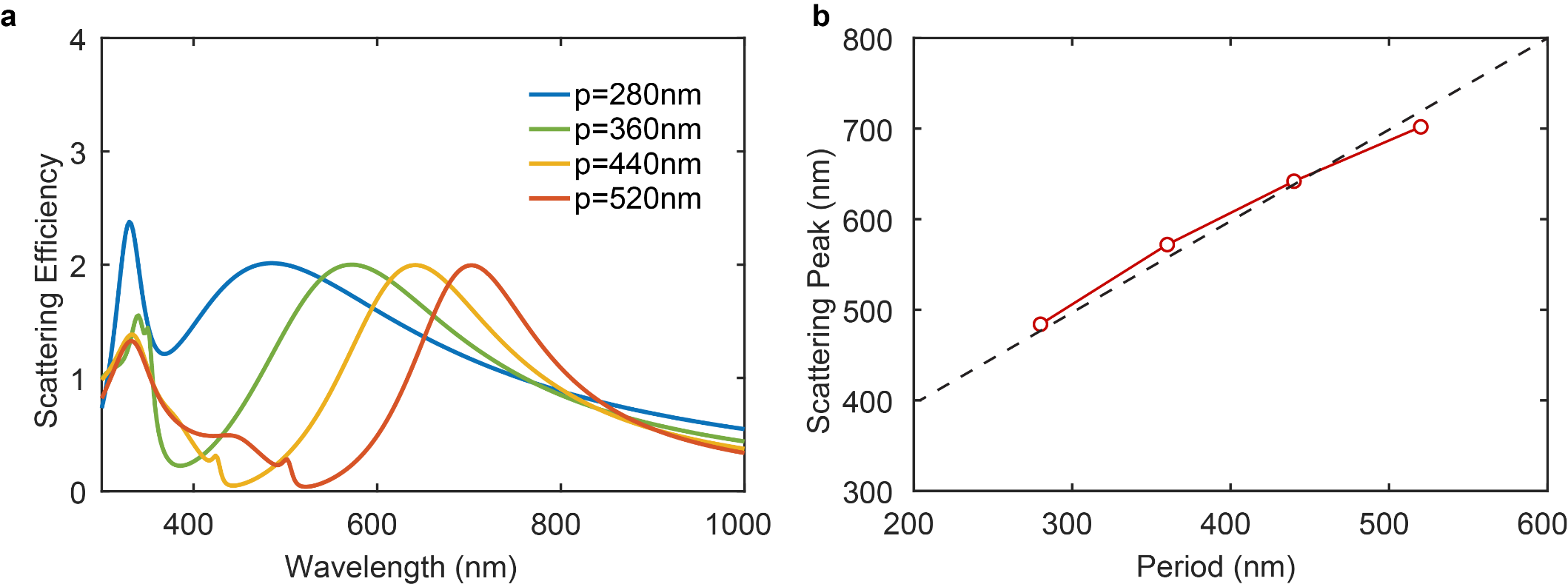


**Supplementary Figure 2.** (a) Simulated scattering efficiency of a Si NW array (*h* = *w* = 55 nm) as a function of the incident wavelength for different array periods *p*. The scattering efficiency is defined as the ratio between the scattering cross section of one period and the physical cross section of one period. (b) The modified first-order resonance position as a function of the period of NW array (red circles) and a linear fit through the data (black dashed line).

**Supplementary Note 3: Multipole decomposition of the scattering by transparent NW array.**

In order to quantitively understand the strong directional scattering/anti-reflection effect in Si NW array, we perform the multipole decomposition calculation [3-5] of the simulated full-fields in order to verify that the transparency is indeed induced by the destructive interference by two optical modes with opposite symmetry. First of all, we notice that the first-order optical resonance supports linear electric dipole moment and second-order optical resonance supports effective linear magnetic dipole moment (rigorously, magnetic dipole is degenerate with electric quadrupole for the 2D case, but they have the same far-field radiation distribution in terms of its linear moment). We perform a multipole decomposition in the cross-sectional plane for one NW unit cell (with unity length in *z*-direction) in a NW array:

$\mathbf{P}\left( \mathbf{r} \right)\cong\mathbf{p}\delta\left( \mathbf{r} \right)-\frac{1}{6}\mathbf{Q}\nabla\delta\left( \mathbf{r} \right)+\frac{i}{\omega}\left[ \nabla\times\mathbf{m}\delta\left( \mathbf{r} \right) \right]$ (11)

$\sim\mathbf{p}\delta\left( \mathbf{r} \right)+\frac{i}{\omega}\left[ \nabla\times\left( \mathbf{m}_{\mathbf{eff}} \right)\delta\left( \mathbf{r} \right) \right]$ (12)

Here:

$\mathbf{p}=\iint\mathbf{P}\left( \mathbf{r}^{\mathbf{'}} \right)dxdydz$ (13)

$\mathbf{m}=-\frac{i\omega}{2}\iint\left[ \mathbf{r}^{\mathbf{'}}\boldsymbol{\times}\mathbf{P}\left( \mathbf{r}^{\mathbf{'}} \right) \right]dxdydz$ (14)

$\mathbf{m}_{\mathbf{eff}}=2\mathbf{m}$ (15)

$\mathbf{P}\left( \mathbf{r}^{\mathbf{'}} \right)=\varepsilon_{0}\left( \varepsilon_{\mathrm{Si}}-1 \right)\mathbf{E}\left( \mathbf{r}^{\mathbf{'}} \right)$ (16)

The scattering cross section of electric dipole (ED) and effective magnetic dipole (MD) are expressed as (ignoring the substrate effect):

${C_{\mathrm{sca}}}^{\left( \mathrm{ed} \right)}=\frac{{k_{0}}^{4}}{6\pi{\varepsilon_{0}}^{2}{E_{0}}^{2}}\left| \mathbf{p} \right|^{2}$ (17)

${C_{\mathrm{sca}}}^{\left( \mathrm{md} \right)}=\frac{{\eta_{0}}^{2}{k_{0}}^{4}}{6\pi{E_{0}}^{2}}\left| \mathbf{m}_{\mathbf{eff}} \right|^{2}$ (18)

For the entire NW array, a two-dimensional dipole lattice gives rise to a pre-factor function added in front of the scattering electromagnetic field:

${{E(H)}_{sca,tot}}^{(ed, md)}=F\cdot{{E\left( H \right)}_{\mathrm{sca}}}^{\left( ed, md \right)}$ (19)

Here

$F\propto\sum_{m, n} \frac{1}{R(m,n)}e^{ik\cdot R(m,n)}\cos\left( \alpha\left( m,n \right) \right)$ (20)

*R* is the distance between the source point and field point and *α* is angle between dipole direction and radiation direction. We thus find:

${C_{sca,tot}}^{\left( ed,md \right)}\propto{{E^{2}\left( H^{2} \right)}_{sca,tot}}^{\left( ed, md \right)}\propto{{{E^{2}\left( H^{2} \right)}_{\mathrm{sca}}}^{\left( ed, md \right)}\propto C_{\mathrm{sca}}}^{\left( ed,md \right)}$ (21)

As a result, the back-scattering intensity for the whole NW array from different dipole components is proportional to the scattering cross section of a unit cell of one NW. We calculate the normalized scattering cross section for the electric dipole and effective magnetic dipole of a unit cell as a function of incident wavelength and the height of the NW array as shown in Supplementary Fig. 3a, b. Supplementary Fig. 3c shows the trend for the scattering cross section peak of electric dipole and effective magnetic dipole component. The error bars show the region where the amplitude of the scattering cross section component is larger than 80% of its peak value. We find that when the height of the NW array is 110 nm, the electric dipole overlaps with effective magnetic dipole around 550 nm, indicating that the degenerate optical resonance is indeed realized. To visualize the destructive interference between electric dipole (first-order optical resonance) and magnetic dipole (second-order optical resonance), we also plot the phasor-diagram of the normalized complex reflected electric field from electric dipole, magnetic dipole, their superposition (back scattering), the bare substrate, and the whole system. In the calculation, *E*_ed(md)_ *= E*_backscattering_*∙E*_sca_^ed(md)^/(*E*_sca_^ed^*+ E*_sca_^md^), and *E*_total_, *E*_substrate_, and *E*_backscattering_ are taken from full field and scattering field simulations.


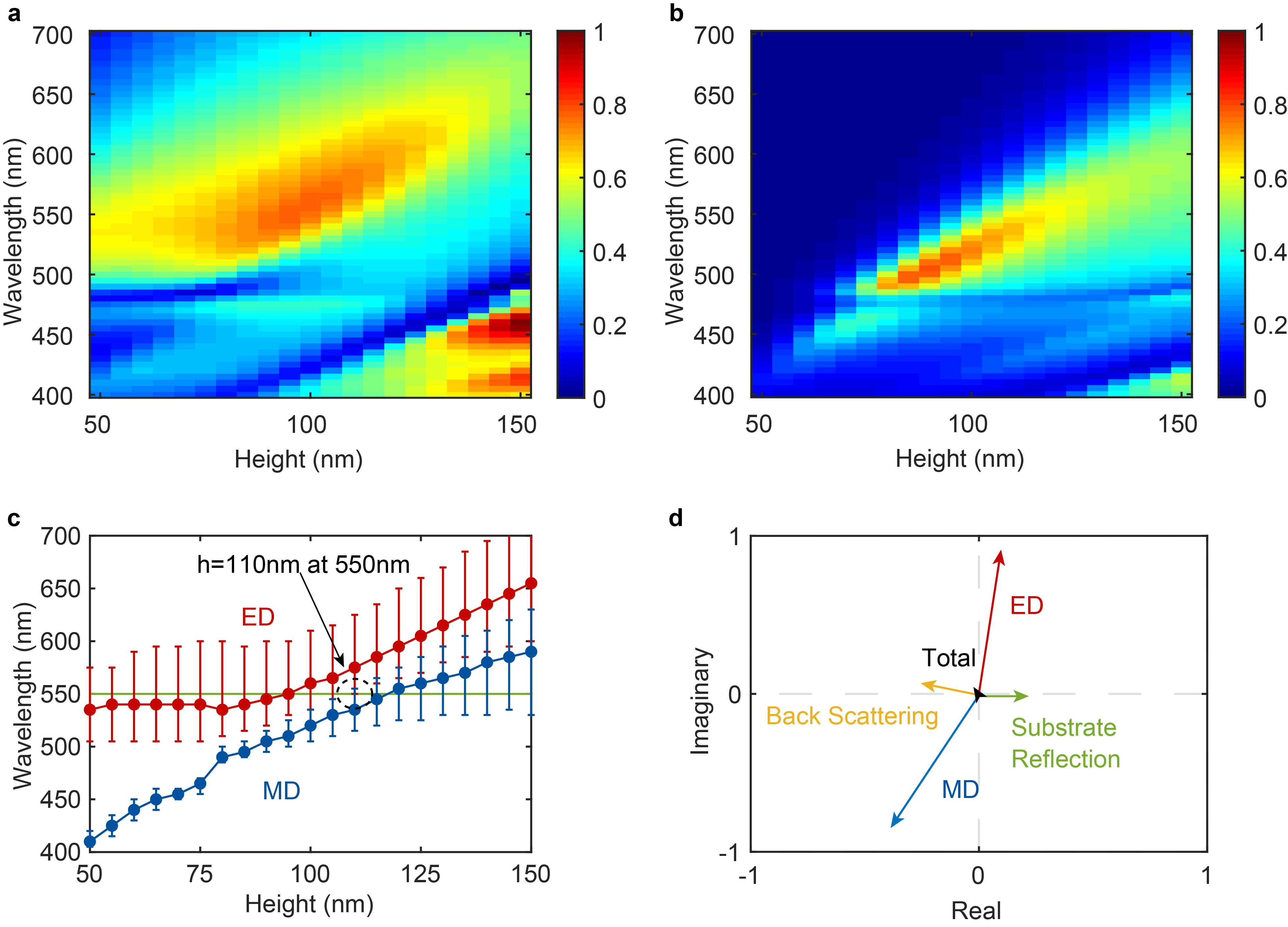


**Supplementary Figure 3.** (a) The normalized scattering cross section from the electric dipole and (b) the effective magnetic dipole component calculated by a multipole decomposition as a function of the height of the NW and the incident wavelength. The width of the NW and its period are fixed as 55 nm and 280 nm, respectively. The sapphire substrate, material dispersion and loss are considered in the full field simulations. (c) The trend of the scattering cross section peak of the electric dipole and effective magnetic dipole component. The error bars show the region where the amplitude of the scattering cross section component is larger than 80% of its peak value. (d) Phasor-diagram of the complex electric field of reflected light from different channels.

**Supplementary Note 4: Comprehensive comparison between “conventional” and “transparent” NW arrays.**

In Fig. 1e, we used relatively narrow-band illumination (center wavelength 550 nm, FWHM = 32 nm) to highlight the contrast between the “conventional (*h* = 55 nm)” and “transparent (*h* = 110 nm)” NW arrays. However, we emphasize that even under the white light illumination, the “transparent” NW array still shows significantly higher transparency under TM polarization (as shown in Supplementary Fig. 4a-c), without suppressing the photodetection performance. On the contrary, the averaged transmittance of the “conventional” NW array is only ~ 30%. Fundamentally, unlike electromagnetic induced transparency (EIT), for the “transparent” array we do not sacrifice broadband transparency to achieve high transparency at a target wavelength. The non-perfect anti-reflection away from 550 nm is due to the mismatch in quality factor between the symmetric and anti-symmetric modes. For the “conventional” array with separated resonant modes, the reflection is significantly larger as no mechanism can be used to suppress the strong back scattering from individual resonant mode.

Additionally, we emphasize that there is no trade-off between EQE and transparency. On the contrary, the EQE (and absorption) is further enhanced by inducing the degenerate optical resonant modes in the “transparent” NW arrays. By assuming that the fabrication processing conditions are the dominant factor determining the Internal Quantum Efficiency (IQE), the “conventional” NW detector should have an IQE similar to the “transparent” NW detector. As a result, the comparison of absorption spectra is sufficient to compare the optoelectronic performance of the two structures. Supplementary Fig. 4d clearly shows that the degenerate optical modes enhance the absorption by a factor 4 compared to the “conventional” detector, and the resulting sharper absorption peak is critical for color detection. A calculated EQE spectra of “conventional” NW detector (by assuming that it has the same IQE as the “transparent” NW detector) is also included in Supplementary Fig. 4e for comparison.

**
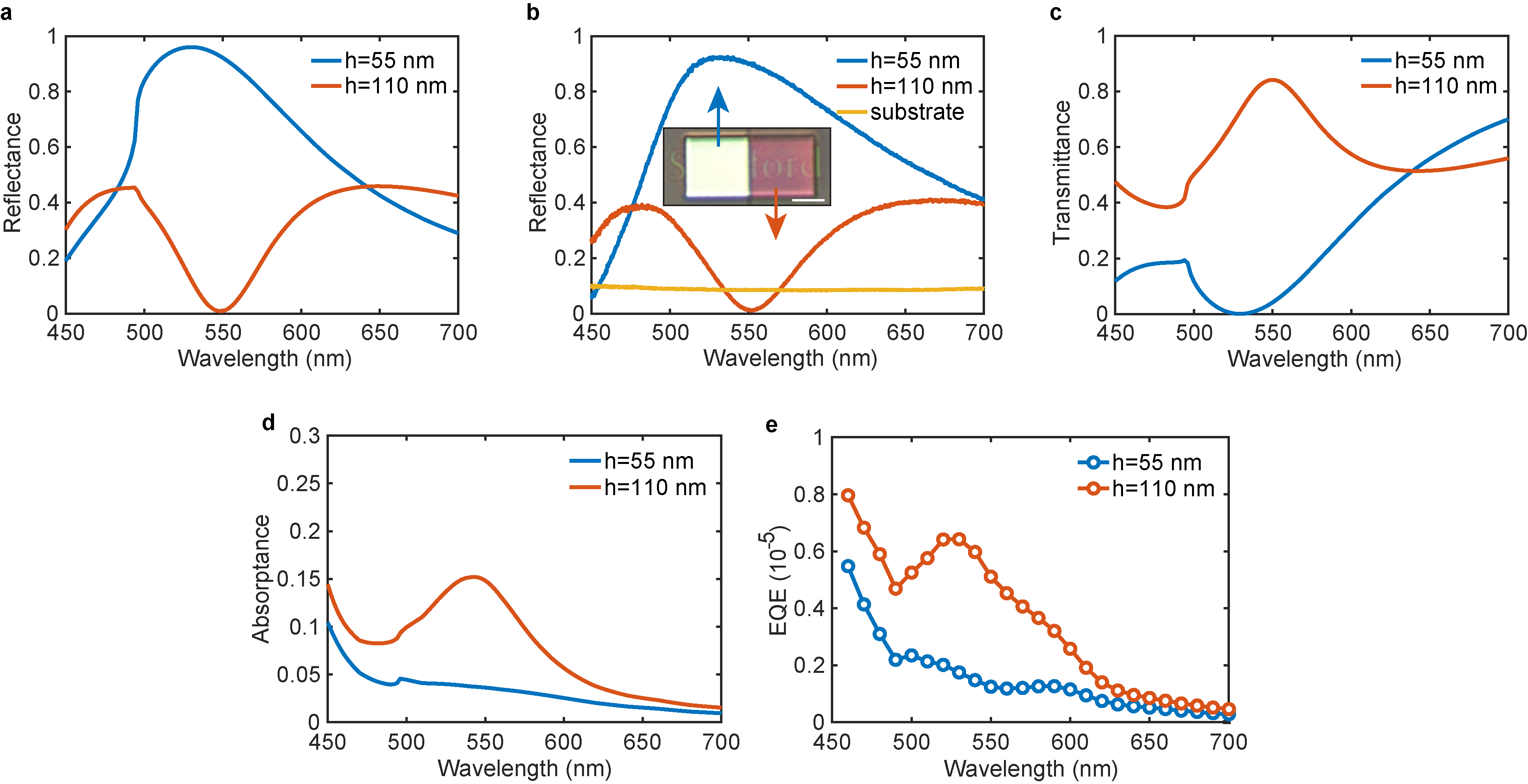
**

**Supplementary Figure 4.** (a) Simulated and (b) measured reflection spectra for “conventional” (*h* = 55 nm) and “transparent” NW arrays. Inset: reflection optical image of “conventional” (left) and “transparent” (right) NW arrays under TM-polarized white light illumination. Scale bar: 25 μm. (c) Simulated transmission and (d) absorption spectra for “conventional” (*h* = 55 nm) and “transparent” NW arrays. (e) Measured (for “transparent” NW detector) and simulated (for “conventional” NW detector) EQE spectra under TM illumination.

**Supplementary Note 5: SEM images of fabricated three NW arrays.**


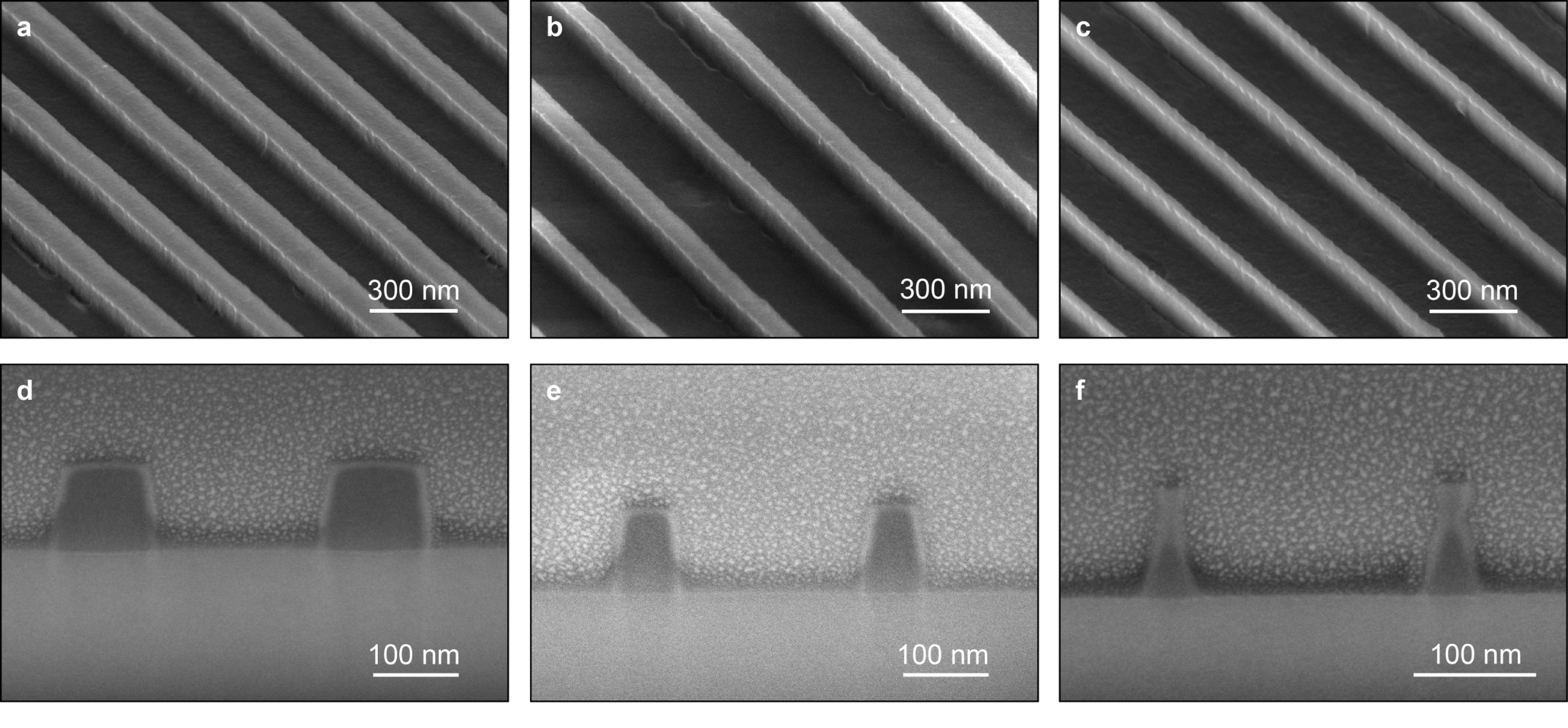


**Supplementary Figure 5.** (a-c) Tilted view SEM images of fabricated “red”, “green”, and “blue” NW arrays, respectively. (d-f) Cross-section SEM images of fabricated “red”, “green”, and “blue” NW arrays, respectively. E-spacer is spin coated to reduce the charging effect and visible in the cross-section SEM images (d-f).

**Supplementary Note 6: Full field simulations of “red”, “green”, and “blue” NW arrays.**


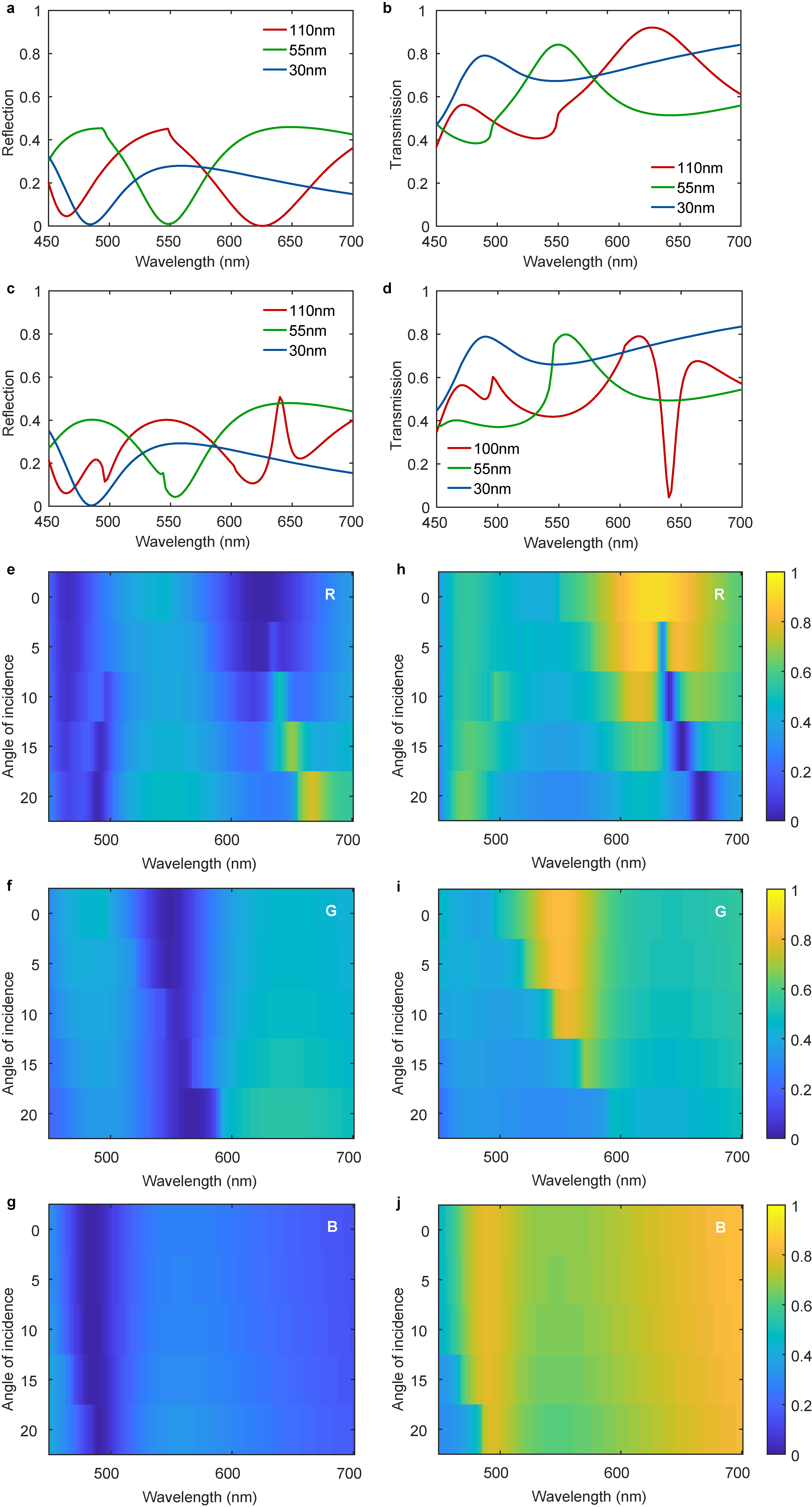


**Supplementary Figure 6.** (a,c) Simulated reflection and (b,d) transmission spectra for the three designed NW arrays for TM polarization as a function of incident wavelength (a,b) under normal illumination and (c,d) inclined illumination with 10° incident angle. The height of all NW arrays is 110 nm. The wire width of the “red”, “green”, “blue” NW array is 110 nm, 55 nm, and 30 nm, with a period of 310 nm, 280 nm, and 230 nm, respectively. The diffraction edges for the “red” and “green” NW arrays can be observed in (a,b) at 550 nm and 496 nm, respectively. (e-g) Simulated reflection and (h-j) transmission map as a function of incident wavelength as well as angle of incidence for “red”, “green”, “blue” NW arrays, respectively.

**Supplementary Note 7: Guided mode resonance in “red” NW arrays.**

In Fig. 2b, a small wiggle occurs around 600 nm for “red” NW array (*w* = 100 nm, *h* = 110 nm, *p* = 310 nm). However, we don’t observe it in the simulations for normal incidence illumination (Supplementary Fig. 5b). This is due to the fact that the excited guided mode resonance here has an anti-symmetric field distribution so that it becomes a “dark” mode under normal incident TM-polarized illumination. The eigen mode calculation is performed with a (lossless) Si NW array suspended in the air as shown in the inset to Supplementary Fig. 6. This mode has a purely real eigenfrequency (infinitely large quality factor), indicating that there is no coupling between the guided mode resonance and free-space light. This “dark” mode is described in details in [6]. Under off-normal illumination, the associated guided mode resonance becomes bright so that we observe a sharp valley in the simulated spectra. In the experiment, a 20× objective lens (NA = 0.4) is used to collect light, giving rise to the observed guided mode resonance excited by off-normal incident light. For “green” and “blue” NW arrays, this mode is blue-shifted to a wavelength shorter than 450 nm, thus we don’t observe them in the experiments.


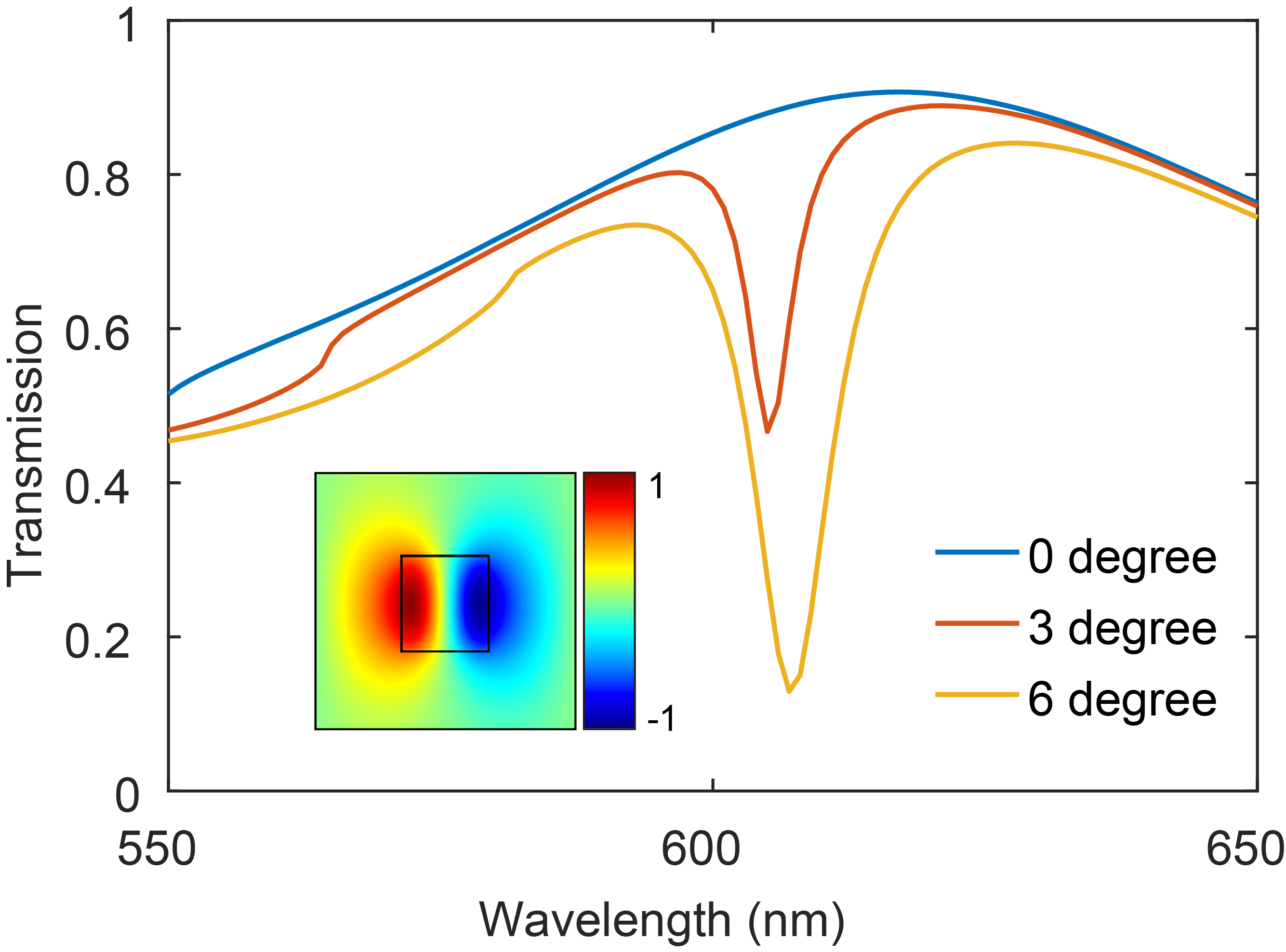


**Supplementary Figure 7.** Transmission of the “red” NW array as a function of incident wavelength for different incident angle. Inset: The electric field distribution (*E*_z_) of the eigen mode of the “dark” guided mode resonance under normal illumination. The resonant wavelength is 596 nm with infinitely large quality factor (no leakage). The eigen mode simulation is conducted with a Si (*n* = 4, lossless) NW array suspended in air.

**Supplementary Note 8: Transparency of three NW arrays for TE polarization.**

In the main text, we mainly focus on the optical properties of Si NW array for TM polarization. For practical purposes, it is essential to show that we also have good transparency for TE polarization and TE-polarized light doesn’t disarrange the color detection. Supplementary Fig. 7 shows the simulated (top) and measured (bottom) reflection and transmission spectra of the three Si NW arrays considered in the main text. We find that the “blue” and “green” NW arrays serve as an almost perfect non-resonant anti-reflection coating at the targeted wavelengths, resulting in excellent transparency with negligible optical absorption. For the “red” NW array, inevitably the TE_11_ optical resonance is excited, leading to a reflection peak around 600 nm. This additional reflection peak does decrease the overall transmission, but thanks to the good transparency for the “blue” and “green” NW arrays, the overall transmission is still ~70% and white-balanced. Moreover, it turns out that the absorption associated with TE_11_ resonance is almost degenerate with TM_11_ resonance (Supplementary Fig. 8a) so that the color detection is still valid for un-polarized light.


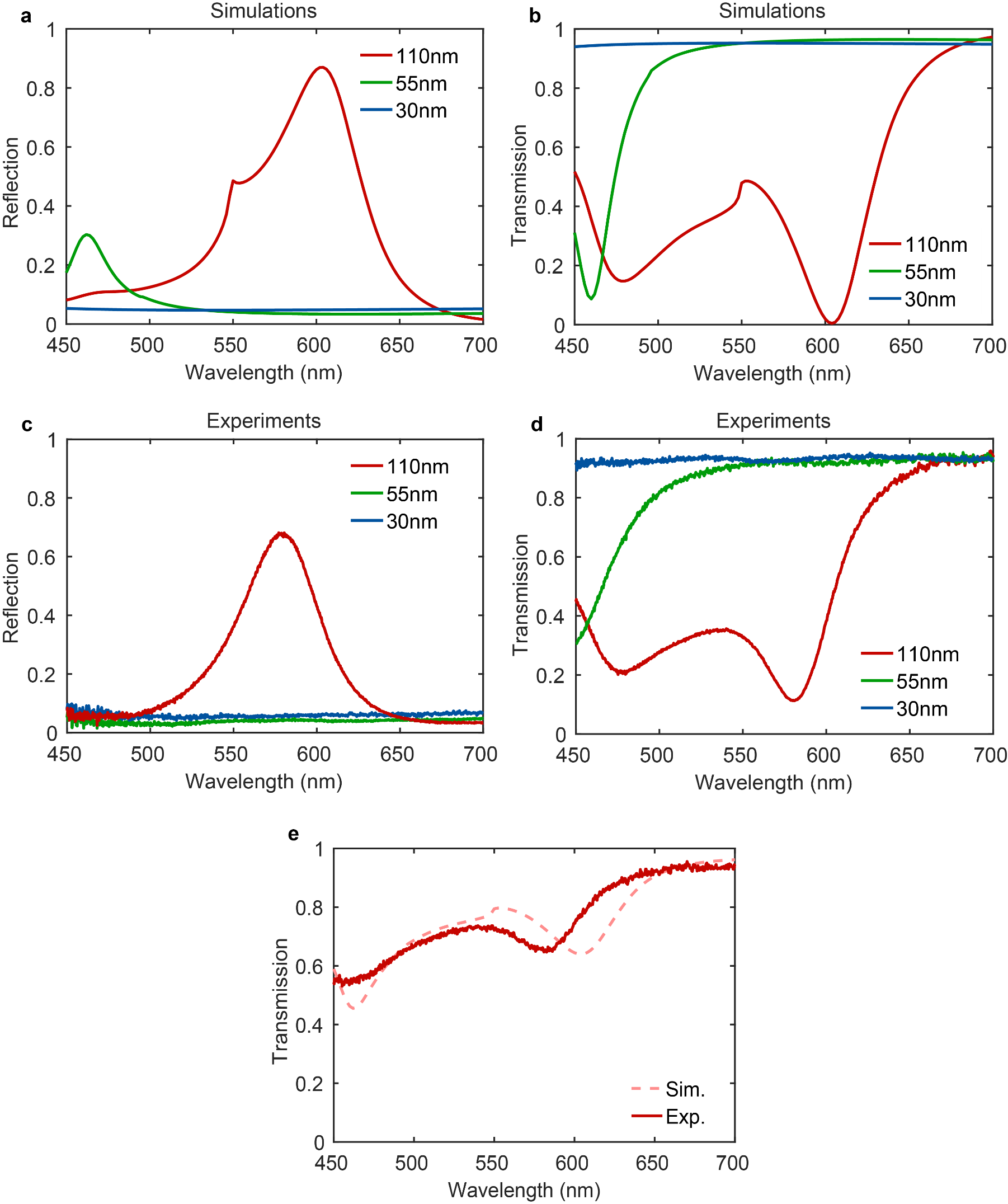


**Supplementary Figure 8.** (a) Simulated reflection and (b) transmission of three designed NW arrays for TE polarization as a function of incident wavelength. (c) Measured reflection and (d) transmission of three designed NW arrays for TE polarization as a function of incident wavelength. (e) Simulated and measured overall transmission as a function of incident wavelength by averaging the transmission spectra of three designed NW arrays.

**Supplementary Note 9: Optoelectronic performance of designed three Si NW photodetectors.**


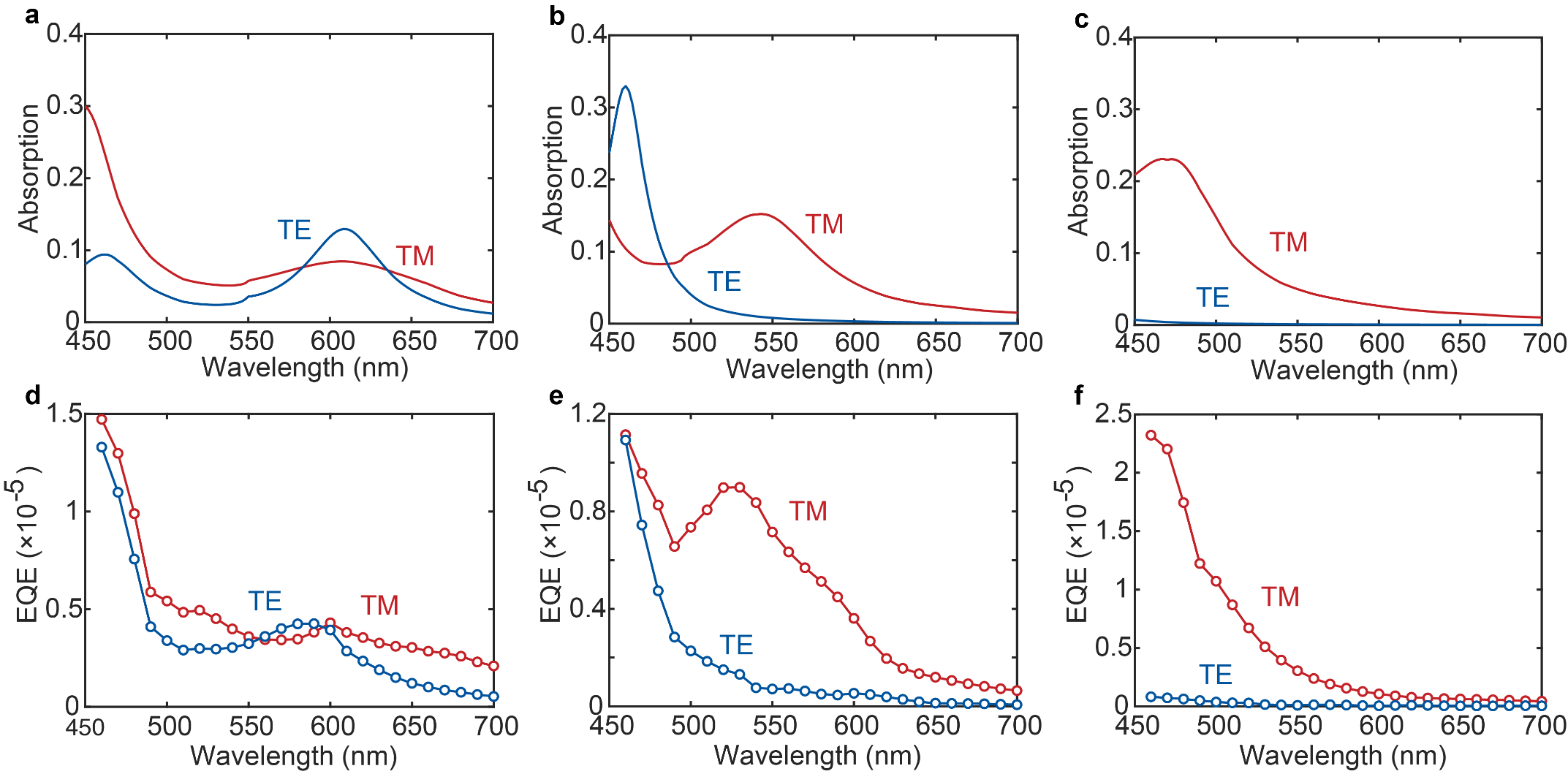


**Supplementary Figure 9.** (a-c) Simulated optical absorption as a function of incident wavelength of the three designed NW photodetectors for both polarizations. (d-f). Measured EQE as a function of incident wavelength of the three fabricated NW photodetectors for both polarizations.

**Supplementary Note 10:** Comparison between the color space generated by the designed photodetectors and the CIE 1931 color space.

The 1931 CIE color space is the first defined quantitative link between distributions of light at different wavelengths in the electromagnetic visible spectrum, and physiologically perceived colors for human color vision. Tristimulus values are defined in such a way that humans see the same color as long as the corresponding tristimulus values calculated from the spectra are the same. Obviously, the adopted tristimulus values should be directly linked to the optical response of three kinds of cone cells that sense light in human eyes through a linear transformation, because the optical response of cone cells is the only channel for humans that afford color recognition.

For any digital color sensors, the ultimate goal is to have an optical response as close to the cone cells in human eye as possible (or directly similar to the spectra of adopted tristimulus values). Thus, the artificial color space should have a similar shape as the standard CIE 1931 color space. As shown in Supplementary Fig. 9a, b, the color space generated by our designed photodetectors does have a similar shape as the CIE 1931 color space. The smaller ratio contrast of our artificial color space can be further optimized by data post-processing, as shown for example in Supplementary Fig. 9c. Similar to what happens in human eyes, we can define the specific linear transformation to fit the artificial color map to the 1931 CIE color space:

$\left( \begin{matrix} R \\ G \\ B \end{matrix} \right)=\left( \begin{matrix} 1.1 & 0 & -0.5 \\ -0.25 & 1.8 & -0.25 \\ -0.3 & 0 & 1.2 \end{matrix} \right)\left( \begin{matrix} I_{R} \\ I_{G} \\ I_{B} \end{matrix} \right)$ (22)


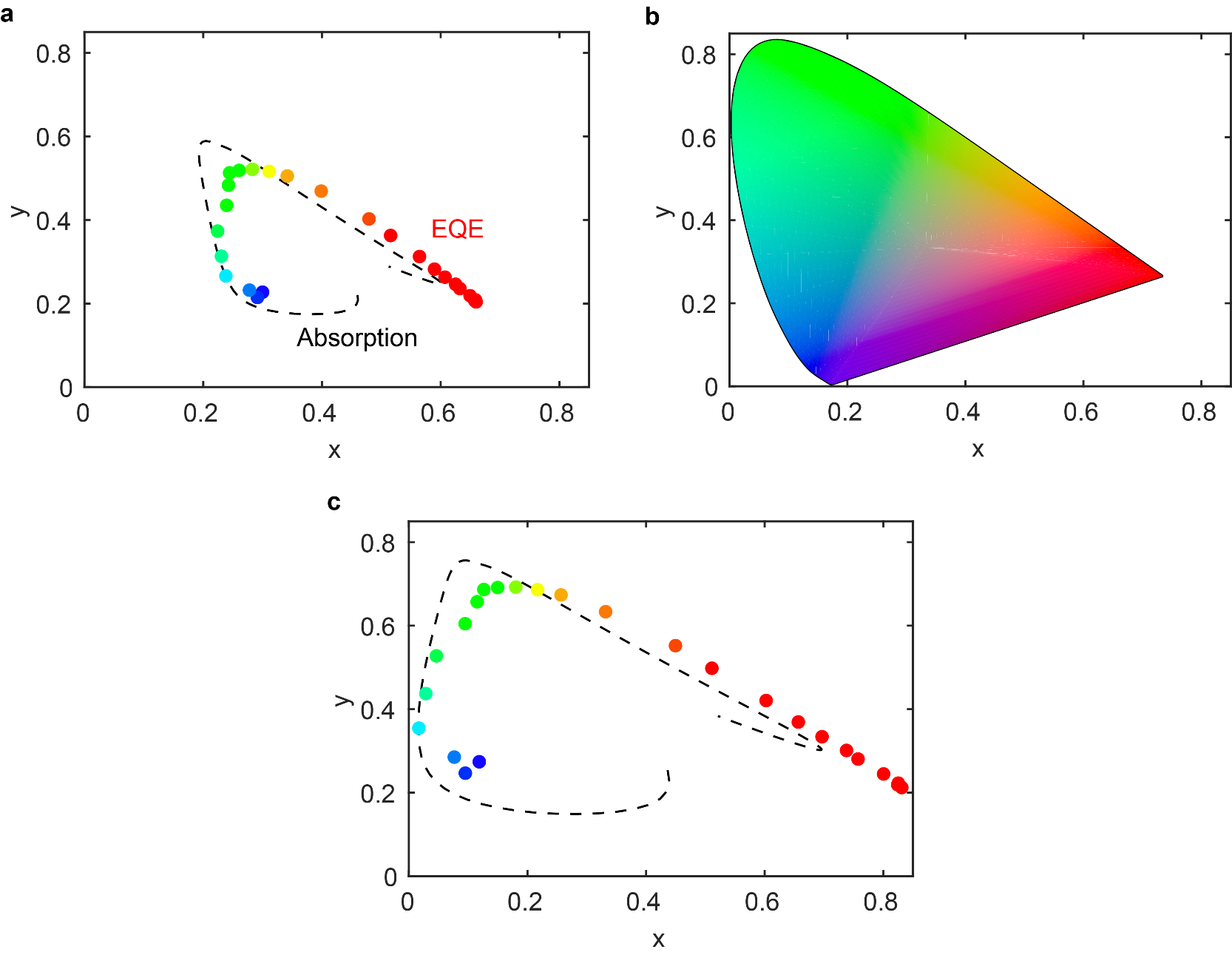


**Supplementary Figure 10.** (a) The color space defined by the extracted photocurrent values from the three fabricated NW array photodetectors. *X*-axis is defined as $I_{R}/(I_{R}+I_{G}+I_{B})$and *y*-axis is defined as $I_{G}/(I_{R}+I_{G}+I_{B})$. (b) The CIE 1931 color space [7, 8]. (c) The optimized color space through the linear transformation. *X*-axis is defined as $R/(R+G+B)$and *y*-axis is defined as $G/(R+G+B)$.

**Supplementary Note 11: Optical properties of Si/ITO interdigitated photodetectors.**

As shown in Supplementary Fig. 10a, b, the Si/ITO interdigitated photodetectors show a near-perfect anti-reflection and an enhanced transmission at three designed wavelengths. It can be understood that these ITO electrodes only provide a small electric dipole component due to their lower refractive index and it can be easily balanced by slightly tuning the geometry of the Si NW array. Supplementary Fig. 10c, d show the corresponding measurement results for the “green” and “red” detectors, which agree very well with the simulated data. The smaller transmission coefficient may be due to the unwanted absorption by the ITO electrodes and the transparency can be improved further by a better ITO deposition and annealing process.


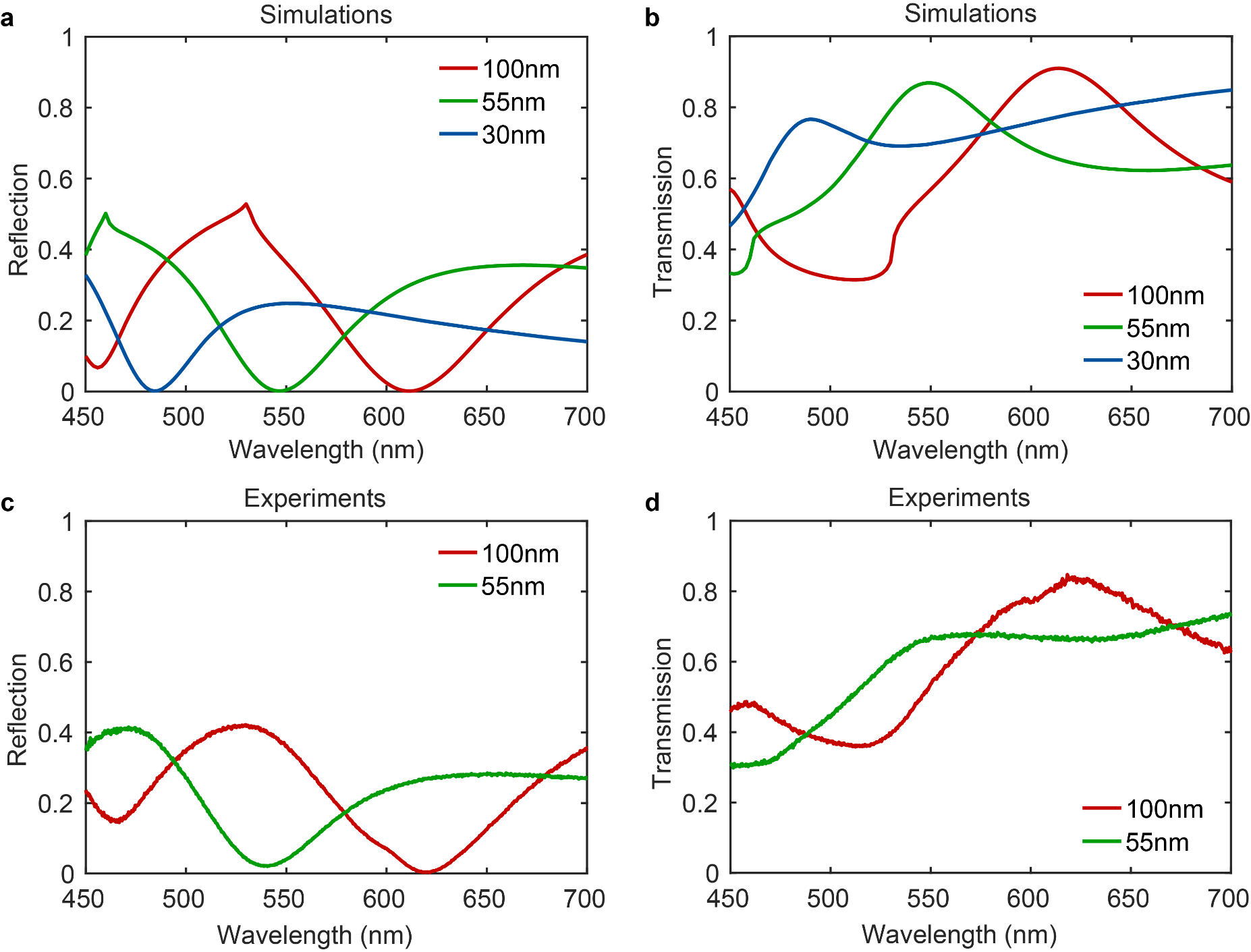


**Supplementary Figure 11.** (a) Simulated reflection and (b) transmission of three designed Si/ITO interdigitated NW arrays for TM polarization as a function of incident wavelength. The height of all NW arrays is 110 nm. The “red”, “green”, and “blue” NWs are 100 nm, 55 nm, and 30 nm wide with a period of 300 nm, 260 nm, and 240 nm, respectively. (c) The measured reflection and (b) transmission of fabricated “red” and “green” Si/ITO interdigitated NW arrays as a function of the incident wavelength*.*

**Supplementary Note 12: SEM images of fabricated “red” and “green” Si/ITO interdigitated NW arrays.**


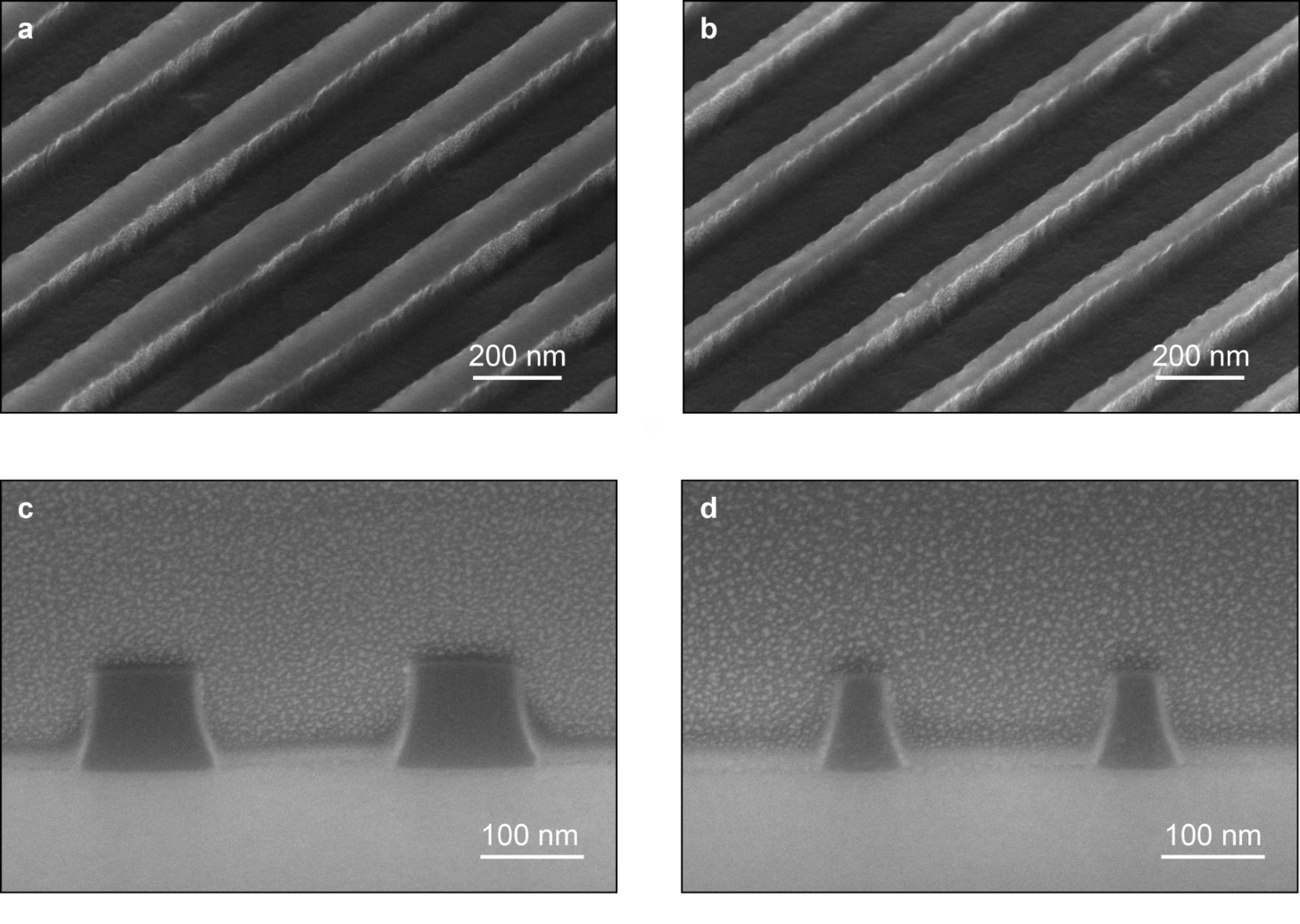


**Supplementary Figure 12.** (a-b) Tilted-view SEM images of fabricated “red”, and “green” Si/ITO interdigitated NW arrays. (d-f) Cross-section SEM images of fabricated “red”, and “green” Si/ITO interdigitated NW arrays, the ITO contact layer is clearly visible. E-spacer is spin coated to reduce the charging effect.

**Supplementary Note 13: Color and polarization detection ­­­­­­­­by Si/ITO interdigitated photodetectors.**

To justify the color and polarization detection ability of Si/ITO interdigitated photodetectors, we simulate the absorption spectra for the “red”, “green”, and “blue” photodetectors for TM polarization as shown in Supplementary Fig. 12a. As we expect, the three detectors mainly absorb red, green, and blue light separately, and the associated color space (shown in Supplementary Fig. 12b) shows that the presence of the ITO electrodes barely changes the absorption spectra for the three photodetectors, giving rise to a similar color detection ability to the bare Si NW array photodetectors. The simulated “red” photodetector optical absorption and measured EQE spectra for two orthogonal polarizations are shown in Supplementary Fig. 12c, d, respectively. They show good agreement and the verified polarization-sensitive optical absorption (inset in Supplementary Fig. 12d) naturally enables the polarization detection.


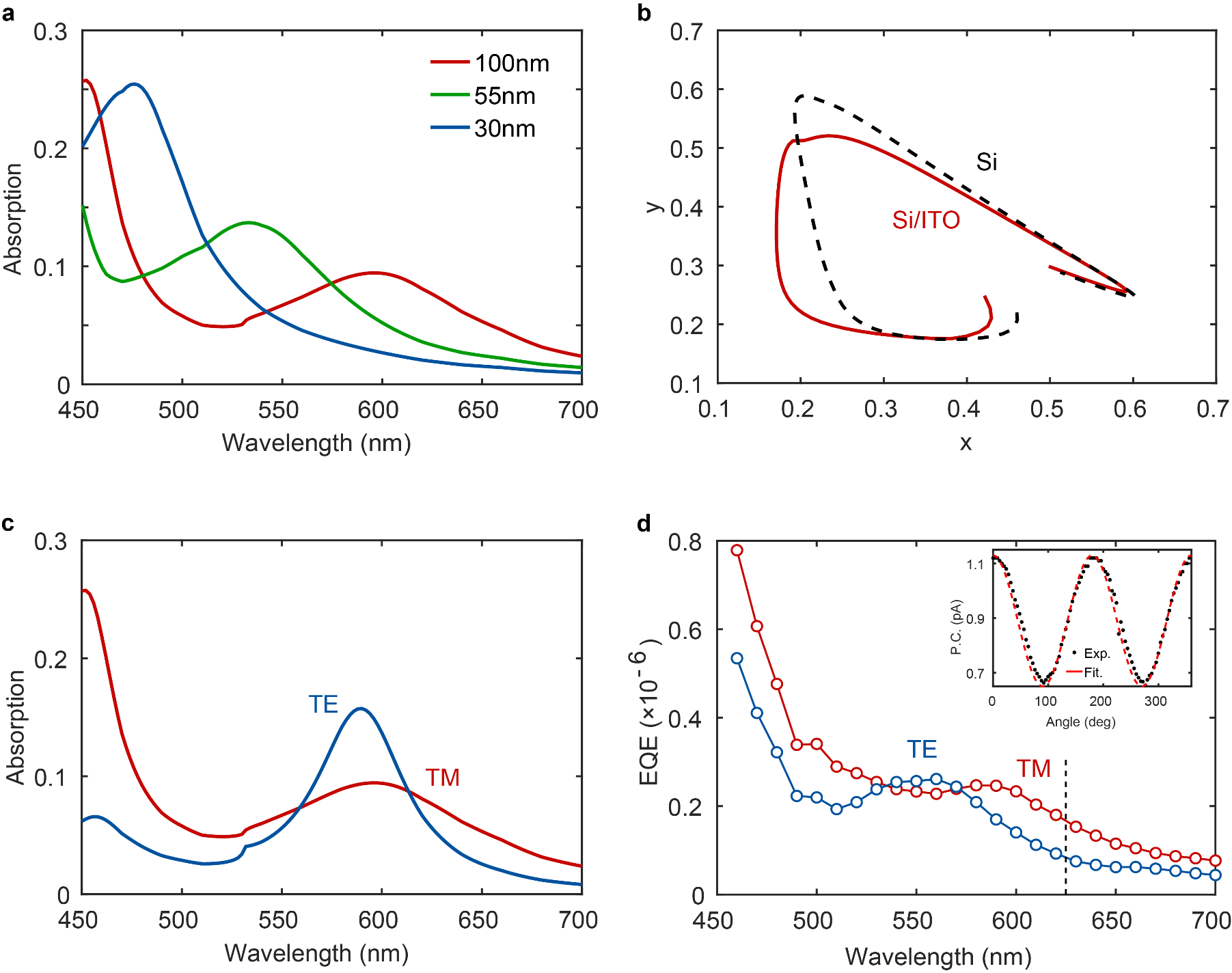


**Supplementary Figure 13.** (a) Simulated optical absorption in three designed Si/ITO interdigitated NW array photodetectors as a function of the incident wavelength. (b) The outlines of the color space defined by the simulated absorption ratio of three Si/ITO interdigitated NW array photodetectors (solid red line) and bare Si NW array photodetectors (dashed black line) under monochromatic illumination from 450 nm to 700 nm. (c) Simulated optical absorption and (d) measured EQE of “red” Si/ITO interdigitated NW array photodetector as a function of incident wavelength for two orthogonal polarizations. Inset: Measured photocurrent at 625 nm (anti-reflection point) as a function of the angle between the incident polarization and the NW direction.

**Supplementary Note 14: Broadening the anti-reflection bandwidth by a multiplexed NW array.**

We should note that the bandwidth of the anti-reflection effect is fundamentally limited by different quality factors of first-order and second-order optical resonance in a NW array. Since the second-order optical resonance is narrower (higher *Q*-factor), the first-order optical resonance dominates when the incident light is off resonance, giving rise to an increase in reflection. One of the solutions to broaden the bandwidth of anti-reflection effect is that we can multiplex two different types of NWs together (big-small-big-small) such that they show second-order optical resonances at different wavelengths, effectively broadening the bandwidth of it. Supplementary Fig. 13 shows the simulated reflection of a multiplexed NW array as a function of the incident wavelength and difference in width of two types of multiplexing NWs. A double-valley reflection spectrum is observed for multiplexed NW arrays. This is attributed to the fact that the “big” and “small” NWs show the second-order optical resonance at different wavelengths. Considering that the first-order optical resonance is broadband, the multiplexed NW array achieves the degenerate optical resonances at two different wavelengths, giving rise to the double-valley feature.


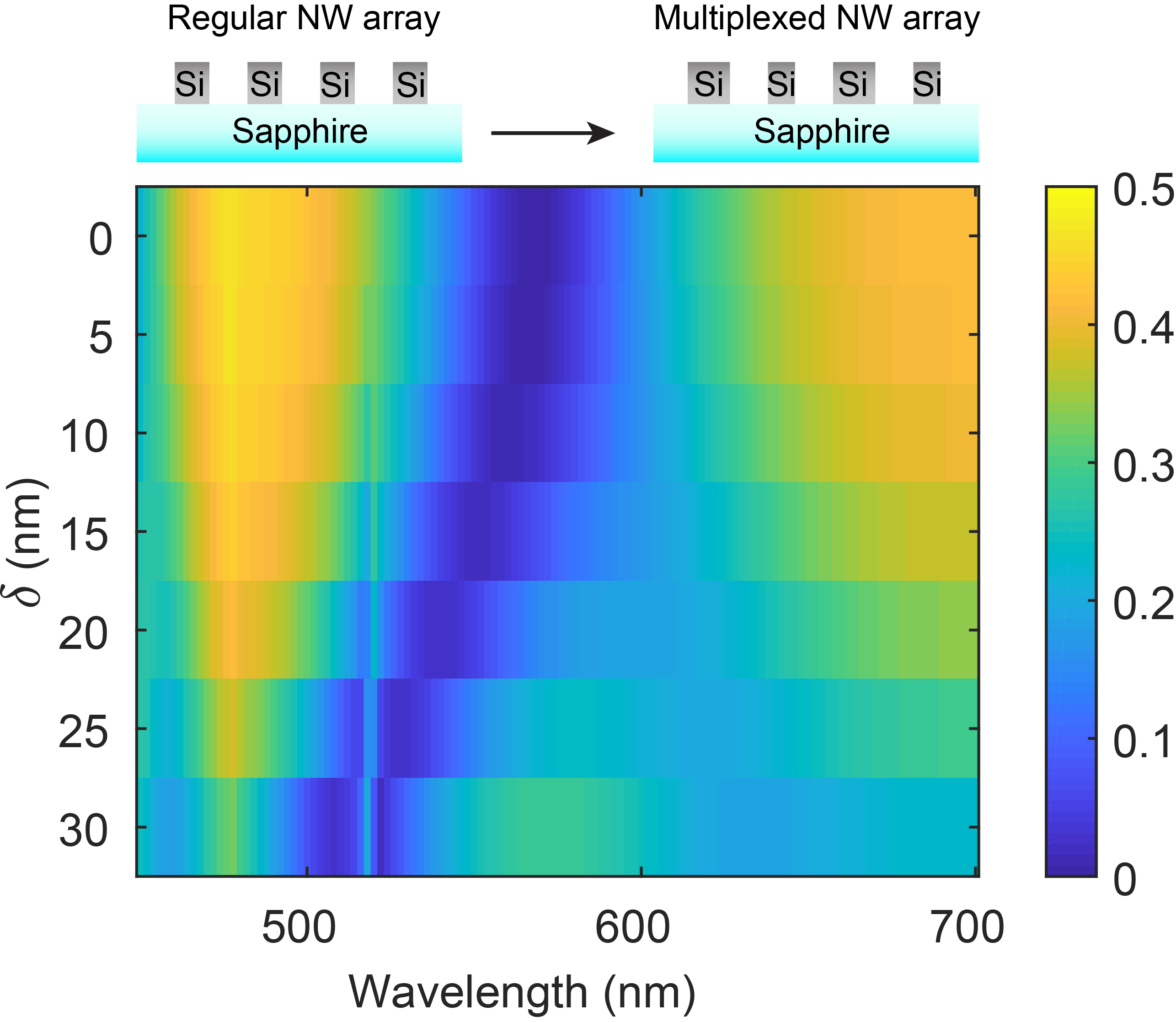


**Supplementary Figure 14.** Simulated reflection of a multiplexed NW array as a function of incident wavelength for different multiplexing elements. The width of the two different NWs in the multiplexed array is 55-$\delta$ nm and 55+$\delta$ nm, respectively. The height of NWs is fixed as 110 nm and the period is 280 nm.

**Supplementary Note 15: Fabrication procedure of Si/ITO interdigitated photodetectors.** ­­­­


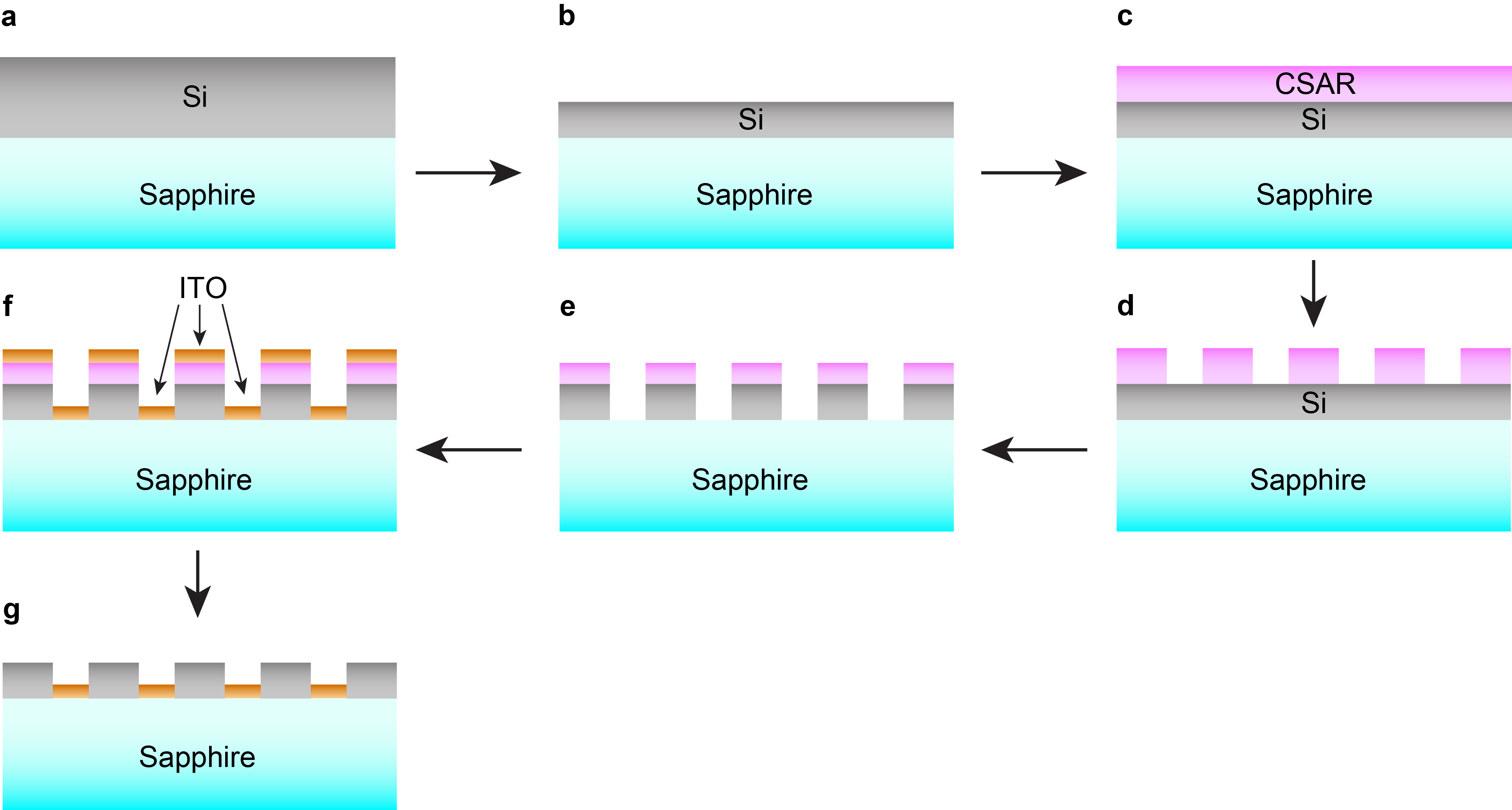


**­­­Supplementary Figure 15.** Schematic of fabrication procedure for Si/ITO interdigitated NW array photodetector. (a) 500 nm-thick Si on sapphire piece. (b) Reactive-ion etching. (c) CSAR Spin coating. (d) Electron-beam exposure and development. (e) Reactive-ion etching. (f) ITO sputtering. (g) Lift-off. See methods for detailed information.

**Supplementary Note 16: Schematic of experiment setup for optical and photocurrent measurement.**


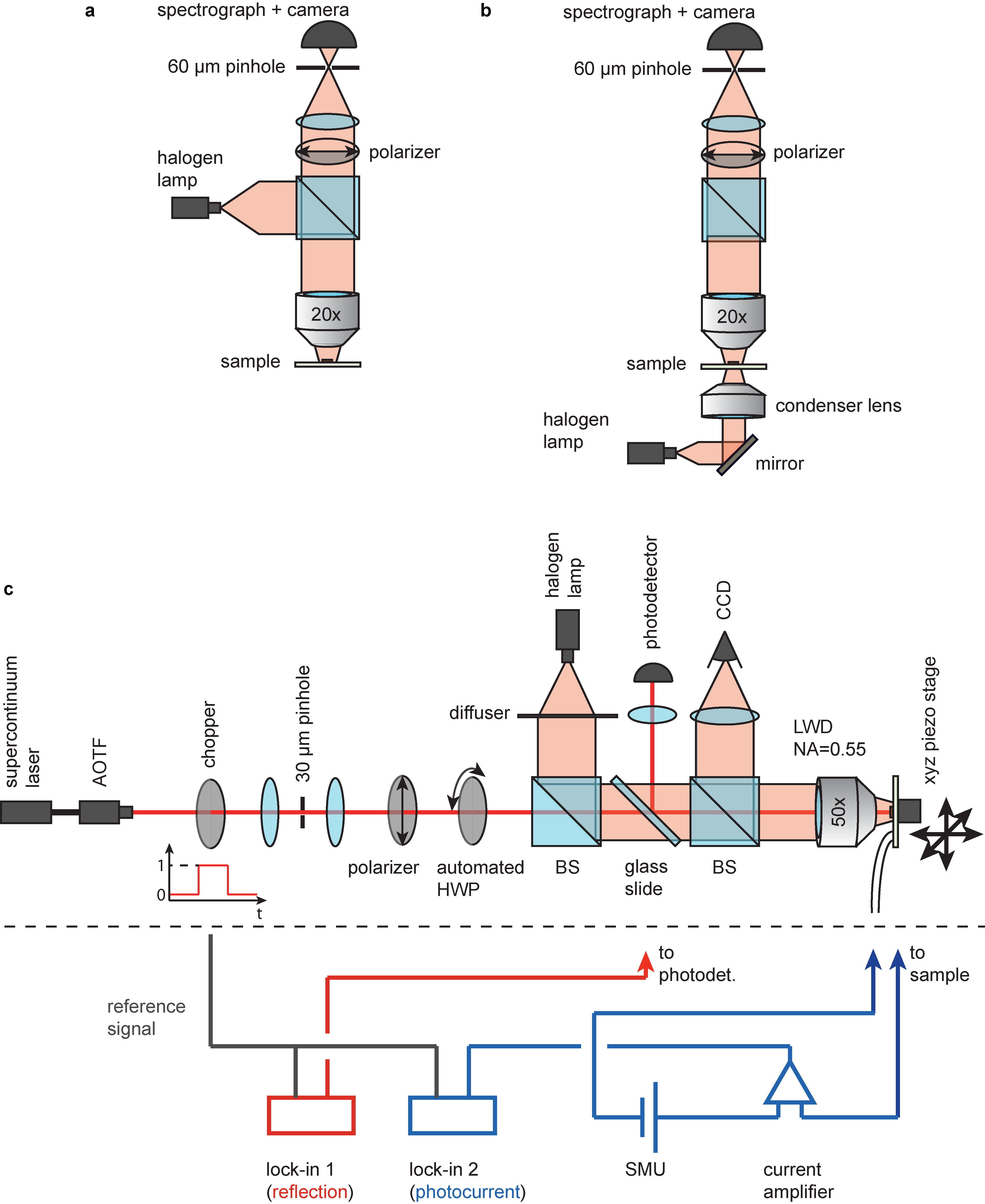


**Supplementary Figure 16.** (a) Schematic of confocal microscope setup used to collect reflection and (b) transmission signal. (c) Schematic of optical (top) and electric (bottom) part of the setup used for photocurrent measurement.

**Supplementary References:**

1. Cao, L. *et al.* Engineering light absorption in semiconductor nanowire devices. *Nat. Mater.* **8**, 643 (2009).
2. Snyder, A. W. Optical Waveguide Theory. *Springer* (1983).
3. Evlyukhin, A. B., Fischer, T., Reinhardt, C. & Chichkov, B. N. Optical theorem and multipole scattering of light by arbitrarily shaped nanoparticles. *Phy. Rev. B* **94**, 205434 (2016).
4. Evlyukhin, A. B., Reinhardt, C., Evlyukhin, E. & Chichkov, B. N. Multipole analysis of light scattering by arbitrary-shaped nanoparticles on a plane surface. *JOSA B* **30**, 2589-2598 (2013).
5. Yu, Y. F. *et al*. High‐transmission dielectric metasurface with 2π phase control at visible wavelengths. *Laser Photonics Rev.* **9**, 412-418 (2015).
6. Vincent, P., Neviere, M. Corrugated dielectric waveguides: A numerical study of the second-order stop bands. *Applied Physics* **20**, 345-351 (1979).
7. Commission internationale de l'Eclairage proceedings. [Cambridge University Press](https://en.wikipedia.org/wiki/Cambridge_University_Press) (1931).
8. Smith, T., Guild, John. The C.I.E. colorimetric standards and their use. *Transactions of the Optical Society* **33**, 73–134 (1931).
